# Oral Administration of *Lactobacillus delbrueckii* during the Suckling Phase Improves Antioxidant Activities and Immune Responses after the Weaning Event in a Piglet Model

**DOI:** 10.1155/2019/6919803

**Published:** 2019-03-03

**Authors:** Yinghui Li, Shuling Hou, Wei Peng, Qian Lin, Fengming Chen, Lingyuan Yang, Fengna Li, Xingguo Huang

**Affiliations:** ^1^College of Animal Science and Technology, Hunan Agricultural University, Changsha Hunan 410128, China; ^2^Hunan Co-Innovation Center of Animal Production Safety, CICAPS, Changsha Hunan 410128, China; ^3^Engineering Research Center for Feed Safety and Efficient Utilization of Education, Changsha Hunan 410128, China; ^4^Institute of Bast Fiber Crops, Chinese Academy of Agricultural Sciences, Changsha, Hunan 410205, China; ^5^Key Laboratory of Agro-Ecological Processes in Subtropical Region, Institute of Subtropical Agriculture, Chinese Academy of Sciences; Hunan Provincial Engineering Research Center for Healthy Livestock and Poultry Production; Scientific Observing and Experimental Station of Animal Nutrition and Feed Science in South-Central, Ministry of Agriculture, Changsha, Hunan 410125, China

## Abstract

Early colonization in the gut by probiotics influences the progressive development and maturity of antioxidant and immune system functionality in the future. This study investigated the impact of orally administrated *Lactobacillus delbrueckii* (LAB) during the suckling phase on future antioxidant and immune responses of the host, using a piglet model. One hundred neonatal piglets received saline (CON) or LAB at the amounts of 1, 2, 3, and 4 mL at 1, 3, 7, and 14 d of age, respectively. The piglets were weaned at the age of 21 d and fed until the age of 49 d. Serum, liver, and intestinal samples were obtained at 21, 28, and 49 d of age. The results showed that LAB tended to decrease serum 8-hydroxy-2-deoxyguanosine concentration and decreased the concentration of serum and hepatic malondialdehyde, but increased the activity of hepatic glutathione peroxidase on days 21, 28, and 49. The concentrations of secretory immunoglobulin A and some inflammatory cytokines and chemokines were increased (*P* < 0.05) in the intestinal mucosa of LAB-treated piglets on days 21, 28, and 49 compared to that of CON piglets. Likewise, protein expression of cyclooxygenase 2 and inducible nitric oxide synthase in the intestine of LAB-treated piglets was increased (*P* < 0.05) during the whole period. These results indicate that administration of LAB to the suckling piglet could improve antioxidant capacity and stimulate intestinal immune response, and these long-lasting effects are also observed up to 4 weeks after weaning. A proper utilization of LAB to neonates would be beneficial to human and animal's future health.

## 1. Introduction

Probiotics are defined as live microorganisms which confer a health benefit on the host when administered in adequate amounts. The beneficial effects of consumption of probiotic products have been attributed to their ability to alleviate lactose intolerance, decreased duration of gastrointestinal infections, and improved digestion and nutritional value of foods. Notably, these beneficial effects also include mitigation of oxidative stress and inflammation modulation, which are associated with normal metabolism but are also involved in the onset of various chronic diseases [1].

Normally, oxidative stress and damage are due to high concentrations of reactive oxygen species (ROS), and the negative effects of which can be balanced by antioxidant defense mechanisms, including antioxidant enzymes such as superoxide dismutase (SOD) and catalase (CAT) as well as nonenzymatic antioxidants such as glutathione [2]. But an abnormality in the antioxidant defense system is known to increase the susceptibility of humans or animals to stress, resulting in inhibited growth and depressed immunity. In several *in vivo* studies, provision of some strains of probiotics has been suggested to improve the oxidative status of rats and humans [3]. However, the mechanisms by which probiotics exert their ability to regulate the antioxidant defense system during physiological processes have not been fully elucidated.

Many probiotic bacteria, especially *Lactobacillus* species which are resident microflora in the gastrointestinal tract of humans and most animals, are able to resist the gastrointestinal environment, to inhibit pathogen load to some extent, and to enhance the immune protection [4]. An increasing number of studies have found that probiotic bacteria could ameliorate inflammation in various disease challenge models [5, 6], for example, through the modulation of inflammatory cytokine production and antimicrobial substance induction [7, 8]. Recently, effectiveness of probiotic administration to human infants at risk of developing gastrointestinal tract diseases has been reported [9]. It seems that neonates are particularly responsive to probiotics due to the immaturity of their immune system and greater simplicity of their intestinal microflora compared with adults. What is more, early administration of probiotics may help develop and maintain microbiota balance during gastrointestinal disturbances [10, 11], which could have a profound influence on the growth potential and health status later in life. Although these findings are encouraging, little is known about how probiotics affect the neonatal immature immune system and whether their influence would be long-lasting.

Therefore, the objective of this study was to use a piglet model to orally administrate *Lactobacillus delbrueckii* (LAB, a gram-positive bacterium belonging to lactic acid bacteria, has traditionally been used in the fermented food industries but repeatedly has been shown to play a beneficial probiotic role nowadays [12]) during the suckling phase and determine the effects of LAB on antioxidant and immune function of preweaning and postweaning piglets. These changes might shed light on the mechanisms mediating the effect of early gut probiotic bacterial colonization on future antioxidant and immune responses of the host.

## 2. Materials and Methods

### 2.1. Bacterial Strain

The strain LAB CCTCC M 207040 used in this study was obtained from the microbiology laboratory of College of Animal Science and Technology, Hunan Agricultural University. In the present study, the viable counts of LAB in culture medium were measured as previously described [13]. In brief, the LAB were incubated in a stationary state at 37°C for 48 h in DeMan, Rogosa and Sharp (MRS) medium in an anaerobic condition. The viable counts in culture medium were determined by the gradient dilution coating method, stored at 4°C, measured and adjusted to 50 × 10^8^ colony-forming units (cfu)/mL, and applied for oral administration to suckling piglets.

### 2.2. Animals and Experimental Design

This study was approved by the animal welfare committee of Hunan Agricultural University. One hundred neonatal piglets (Duroc × landrace × large Yorkshire) from 10 litters (10 piglets per litter) were randomly assigned to 2 groups on the basis of litter origin and body weight (BW), 50 suckling piglets in each group. The control (CON) group of piglets was orally administrated with sterilizing saline, whereas the other group of piglets was orally administrated with LAB. The piglets received the sterilizing saline or the LAB at amounts of 1, 2, 3, and 4 mL per animal at 1, 3, 7, and 14 d of age, respectively. All piglets were housed in an environmentally controlled farrowing cage with hard plastic slatted flooring and had free access to sow milk and drinking water. Piglets were weaned at 21 d of age. Thirty piglets with similar weaning BW were selected from the CON and the LAB groups, respectively, and randomly allotted to 6 replications (pens) of 5 pigs per replicate pen. A total of 60 weaning piglets were fed with creep feed and managed until the age of 49 d. The creep diet was corn-soybean meal-based (52.99% corn, 10.00% extruded corn, 20.00% soybean meal) and contained 14.32 MJ/kg digestible energy and 19.00% crude protein. The diet was formulated as a powder form without any in-feed antibiotics. Piglets were housed in a temperature-controlled nursery and had ad libitum access to feed and water.

### 2.3. Sample Collection

At 21, 28, and 49 d of age, six piglets (1 per replicate) were randomly selected from each treatment for blood and tissue sampling. Blood samples were collected into 10 mL tubes by the venipuncture method from the jugular vein and centrifuged at 3000 × *g* for 15 min at 4°C, and the supernatants (serum) were collected and stored at −80°C for subsequent analysis. Piglets were euthanized with an overdose of sodium pentobarbital solution (40 mg/kg BW) followed by exsanguination. Livers were collected and packaged in sterilized bags, sealed immediately, and frozen quickly in liquid nitrogen. The samples were later stored in a refrigerator at -80°C for analysis of antioxidant indices. The small intestine was dissected free of the mesentery and sampled on a chilled stainless steel tray. Segments (8 cm) of the jejunum and ileum were thoroughly flushed with ice-cold phosphate-buffered saline and divided into two sections. One (approximately 2 cm) segment was immediately frozen in liquid nitrogen and then stored at -80°C for Western blot analysis; the other was used for collecting the mucosa. The mucosal cell layers were scraped off and rapidly frozen in liquid nitrogen and stored at −80°C for the analysis of secretory immunoglobulin A (sIgA), cytokines, and chemokines.

### 2.4. Serum Metabolites, Hormones, and Oxidative Damage Product

The concentrations of serum biochemical parameters, including total protein (TP), albumin (ALB), globulin (GLB), urea nitrogen (UN), glucose (GLU), and alkaline phosphatase (ALP), were measured using the biochemical analytical instrument (Beckman CX4 Chemistry Analyzer; Beckman Coulter Inc., Brea, CA, USA) and commercial kits (Sino-German Beijing Leadman Biotech Ltd., Beijing, China). The concentrations of cortisol and norepinephrine were analyzed with corresponding commercial ELISA kits (Cusabio Biotech Co. Ltd., Wuhan, China) following the recommended procedures. The oxidative damage product 8-hydroxy-2-deoxyguanosine (8-OHdG) was determined using ELISA kits in accordance with the manufacturer's instruction (Cloud-Clone Co., Houston, TX, USA).

### 2.5. Determination of Antioxidant Indices

The antioxidant capacities, including total antioxidant capacity (T-AOC), SOD, glutathione peroxidase (GSH-Px), CAT, and malondialdehyde (MDA) of the serum and liver, were determined using assay kits according to the manufacturer's instructions (Nanjing Jiancheng Bioengineering Institute, Nanjing, China). Protein concentration in the liver was determined following the manufacturer's instructions (bicinchoninic acid assay; Beyotime Biotechnology, Beijing, China). Hepatic antioxidant indices were standardized to the protein in each sample.

### 2.6. Analysis of Intestinal Mucosal sIgA, Cytokines, and Chemokines

The intestinal mucosa was homogenized in ice-cold phosphate-buffered saline and then centrifuged at 10000 × *g* for 10 min at 4°C. The supernatant was collected for measuring sIgA, cytokines, chemokines, and protein contents. Intestinal mucosal sIgA and cytokines, including interleukin- (IL-) 1*β*, IL-6, and IL-12, were measured using ELISA kits in accordance with the manufacturer's instructions (Cusabio Biotech Co. Ltd., Wuhan, China). Intestinal mucosal chemokines, including fractalkine (CX3CL1) and macrophage inflammatory protein 3 alpha (MIP3*α*), were determined using ELISA kits (Abcam, Cambridge, LON, UK, and Cloud-Clone Co., Houston, TX, USA, respectively) according to the manufacturer's instructions. Protein concentration was determined using the bicinchoninic acid assay (Beyotime Biotechnology). Intestinal mucosal sIgA, cytokines, and chemokines were standardized to the protein in each sample.

### 2.7. Western Blot Analysis

The protein expression of cyclooxygenase 2 (COX2) and inducible nitric oxide synthase (iNOS) in the jejunum and ileum was determined by the Western blot technique as we described previously [14]. The following antibodies were used for protein quantification: COX2 (1 : 1000; Abcam, Cambridge, LON, UK), iNOS (1 : 200; Abcam, Cambridge, LON, UK), and *β*-actin (1 : 4000; Proteintech Group Inc.) and secondary antibody horseradish peroxidase-conjugated goat anti-rabbit IgG (1 : 6000; Proteintech Group Inc.) or anti-mouse IgG (1 : 4000; Proteintech Group Inc.). All protein measurements were normalized to *β*-actin, and data are expressed relative to the values in CON piglets.

### 2.8. Statistical Analysis

The results were presented as the mean ± standard error of the mean (SEM). All statistical analyses were performed by IBM SPSS Statistics 22 (IBM Corporation, New York, USA). The statistical analysis between the two groups was performed by Student's *t*-test. Mean values were considered to be significantly different when *P* < 0.05 and were considered to have tendency when 0.05 ≤ *P* < 0.10.

## 3. Results

### 3.1. Serum Biochemical Parameters

The effects of oral administration of LAB during the suckling period on serum biochemical metabolites of the piglets are listed in Table 1. On days 21 and 28, piglets in the LAB group had lower (*P* < 0.05) concentration of UN than those in the CON group. Although no significant differences were observed in the UN concentration between the CON group and the LAB group on day 49, the concentration of UN was 8.8% lower in piglets orally administrated with LAB than in CON piglets. Additionally, on day 49, in comparison with the CON group, the LAB group exhibited an approximate 34.3% increase (*P* < 0.01) in ALP concentration, but it was not obviously different on days 21 and 28. Other serum biochemical parameters including TP, ALB, GLB, and GLU were not significantly changed by LAB throughout the study.

### 3.2. Serum Stress Hormones and Oxidative Damage Product

**Figure 1:**
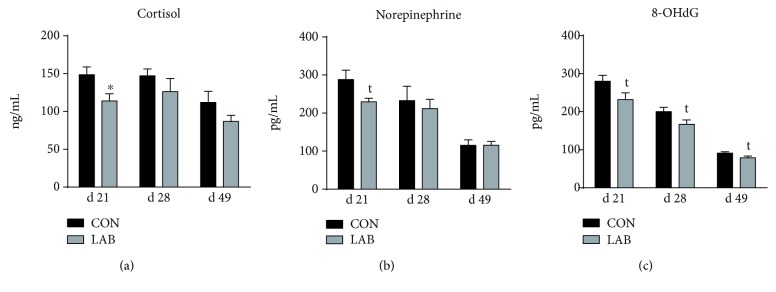
Effects of orally administrated *Lactobacillus delbrueckii* on stress hormones and 8-OHdG of piglets. (a) Serum cortisol concentration in the CON group and LAB group (*n* = 6). (b) Serum norepinephrine concentration in the CON group and LAB group (*n* = 6). (c) Serum 8-OHdG concentration in the CON group and LAB group (*n* = 6). CON = piglets in the negative control group; LAB = piglets in the group that was orally administrated with *Lactobacillus delbrueckii* during the suckling period. Statistical notes refer to differences between the two groups (^∗^*P* < 0.05; *t*: 0.05 ≤ *P* < 0.10).

The serum concentrations of stress hormones (cortisol and norepinephrine) and a biomarker of oxidative DNA damage (8-OHdG) in the piglets were determined at 21, 28, and 49 d of age (Figure 1). On day 21, LAB-given piglets had lower (*P* < 0.05) concentration of cortisol and a trend towards reduced (*P* = 0.06) concentration of norepinephrine compared to the CON piglets, but these evident effects were not observed on days 28 and 49. Furthermore, compared with the CON group, the serum concentration of 8-OHdG had a reducing trend (*P* = 0.07) in the LAB group during the whole period.

### 3.3. Antioxidant Activities

Tables 2 and 3 summarize the effects of oral administration of LAB on antioxidant activities of the serum and liver, respectively. As shown in Table 2, compared with the CON group, the activities of serum T-AOC and CAT increased (*P* < 0.05 and *P* = 0.09, respectively) on day 21 and the activity of CAT was markedly elevated (*P* < 0.01) on day 49 in the LAB group as well. In contrast, the serum MDA concentration of the LAB group on days 21, 28, and 49 was lower than that of the CON group by 36.5% (*P* < 0.05), 30.8% (*P* = 0.06), and 48.7% (*P* < 0.01), respectively. Similar to the serum MDA, the concentration of hepatic MDA was also decreased (*P* < 0.05) in the LAB group compared to the CON group throughout the study (Table 3). Oral LAB administration increased (*P* < 0.05) the activities of hepatic GSH-Px on days 21, 28, and 49 and hepatic CAT on day 21, compared with those of CON. However, the activities of serum SOD and GSH-Px and hepatic T-AOC and SOD were not significantly altered by LAB treatment during the whole period.

### 3.4. Intestinal Mucosal sIgA, Cytokines, and Chemokines

**Figure 2:**
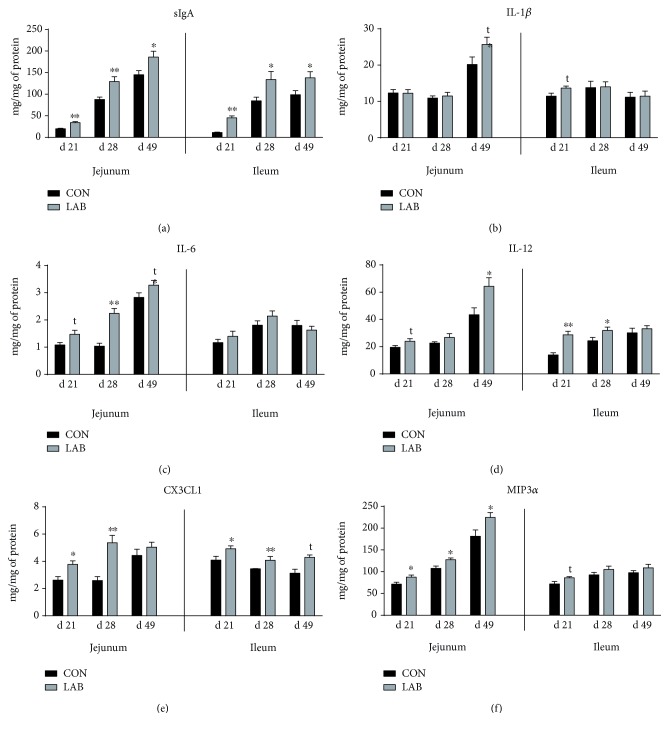
Effects of orally administrated *Lactobacillus delbrueckii* on intestinal mucosal sIgA, cytokines, and chemokines of piglets. (a) sIgA concentration of the jejunal and ileal mucosa in the CON group and LAB group (*n* = 6). (b–d) Cytokine concentrations of the jejunal and ileal mucosa in the CON group and LAB group (*n* = 6). (e–f) Chemokine concentrations of the jejunal and ileal mucosa in the CON group and LAB group (*n* = 6). CON = piglets in the negative control group; LAB = piglets in the group that was orally administrated with *Lactobacillus delbrueckii* during the suckling period. Statistical notes refer to differences between the two groups (^∗∗^*P* < 0.01; ^∗^*P* < 0.05; *t*: 0.05 ≤ *P* < 0.10).

The sIgA concentration of both the jejunum and ileum on days 21, 28, and 49 was higher (*P* < 0.05) in piglets orally administrated with LAB compared with untreated piglets (Figure 2(a)). Additionally, oral LAB administration tended to increase the concentrations of jejunal IL-6 (*P* = 0.06), jejunal IL-12 (*P* = 0.08), and ileal IL-1*β* (*P* = 0.09) on day 21 and jejunal IL-1*β* (*P* = 0.08) and jejunal IL-6 (*P* = 0.09) on day 49 and increased ileal IL-12 (*P* < 0.05) on day 21, jejunal IL-6 and ileal IL-12 on day 28, and jejunal IL-12 on day 49 compared to the concentrations in CON piglets (Figures 2(b)–2(d)). Likewise, concentrations of jejunal and ileal CX3CL1 on days 21 and 28 and jejunal MIP3*α* on days 21, 28, and 49 were increased (*P* < 0.05), and concentrations of ileal MIP3*α* (*P* = 0.05) on day 21 and ileal CX3CL1 (*P* = 0.09) on day 49 also tended to increase in the LAB group compared to the CON group (Figures 2(e) and 2(f)).

### 3.5. Protein Expression of COX2 and iNOS

**Figure 3:**
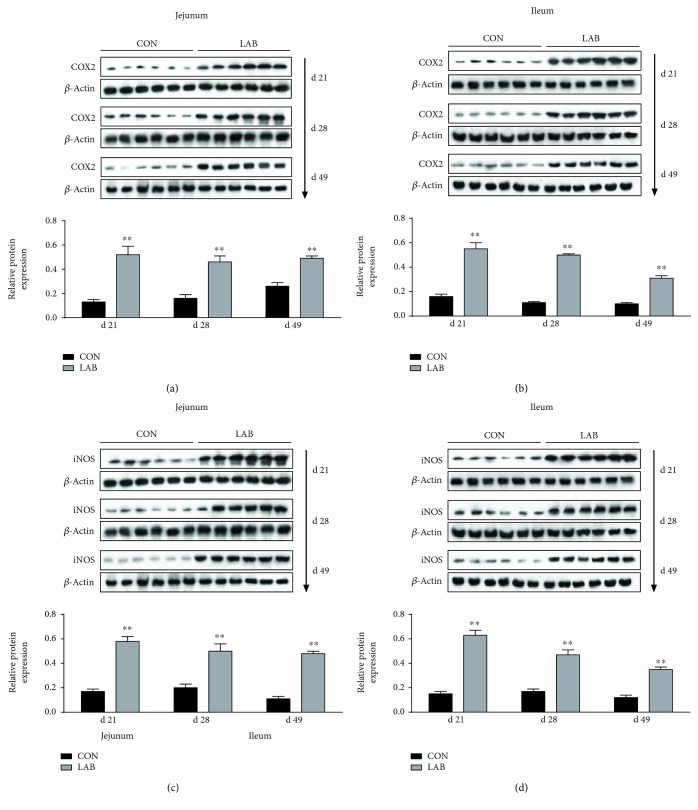
Effects of orally administrated *Lactobacillus delbrueckii* on the protein expression of COX2 and iNOS in the jejunum and ileum of piglets. (a) Protein expression of COX2 in the jejunum of the CON group and LAB group (*n* = 6). (b) Protein expression of COX2 in the ileum of the CON group and LAB group (*n* = 6). (c) Protein expression of iNOS in the jejunum of the CON group and LAB group (*n* = 6). (d) Protein expression of iNOS in the ileum of the CON group and LAB group (*n* = 6). CON = piglets in the negative control group; LAB = piglets in the group that was orally administrated with *Lactobacillus delbrueckii* during the suckling period. Statistical notes refer to differences between the two groups (^∗∗^*P* < 0.01).

The protein expression levels of COX2 and iNOS in the jejunum and ileum are presented in Figure 3. No matter what day it is (days 21, 28, or 49), the protein expression levels of COX2 and iNOS in both the jejunum and ileum in the LAB group were all significantly higher (*P* < 0.01) than those in the CON group.

## 4. Discussion

During the suckling period, milk and the maternal environment shape the gut microbiota of humans and animals such as piglets. However, the composition of microflora is not definitive. It develops gradually, and a transient decrease in favorable bacteria occurs during weaning; the oral supply of probiotics contributes to the reestablishment of the gut microbiota balance [15]. Currently, a growing number of researches indicate that early gut colonization with beneficial microorganisms could produce positive effects on the suckling piglets: promotion of growth performance, reduction of infectious diarrhea, drop in mortality rate, and improvement of intestinal maturity [16–18]. Furthermore, it is increasingly acknowledged that *Lactobacilli* are one of the first colonizers in the gastrointestinal tracts of humans and piglets and contribute many of the above-mentioned benefits [15]. However, our understanding on how *Lactobacilli* affect the reactivity of the antioxidant defense system and immune system later in life is still incomplete. Therefore, in this study, we utilized LAB administrated early in life to the suckling piglet to evaluate its influence on antioxidant capacity and immune function prior to weaning and, most of all, to explore whether it could have a lasting effect after weaning.

Curiously, in terms of serum biochemical parameters, we found that the effects of oral administration of LAB during the suckling phase on serum ALP concentration on day 49 were not highlighted on days 21 and 28. Alkaline phosphatase, an enzyme that catalyzes multiple lecithoid compounds to release inorganic phosphorus, can be deemed as the available index in monitoring the formation of the skeleton and fat. Normally, the activity of serum ALP changes in response to the organism metabolism and animal growth [19]. Our results showed that the activity of ALP was higher in the LAB-treated group, in line with other's findings [20], but its mode of action needs to be further studied. In addition, we noticed that UN, the main and ultimate nitrogenous product of protein catabolism, was decreased during preweaning and postweaning periods when LAB was orally administrated to suckling piglets. This indicates that LAB administration either increases nitrogen use efficiency or decreases protein breakdown, to some extent, reflecting the improved protein catabolism distribution and body load [21]. The alterations in UN are usually influenced by numerous factors, including anabolism-promoting factors and catabolism-stimulating factors such as shock or stress [22]. Both external and internal biological changes such as diet, infection, and psychological events are well documented stressors for piglets, and concurrently, the secretion of cortisol could be escalated under such stressors [23]. Consistent with the UN concentration decline that we recorded, here we observed that the LAB administration lowered the cortisol concentration as well, especially pronounced on day 21, which agrees with the previous report that there is a positive correlation between serum UN and serum cortisol concentrations [24]. It seems likely that the reduction of UN concentration may be partly caused by the decreased concentration of cortisol in association with LAB administration. What is more, our results also suggest that LAB may mitigate the effects of stress and thereby allow piglets to perform at a higher level.

Over the past few years, the in vitro ability of *Lactobacillus* to quench free radicals has been reported [25]. In an evaluation experiment in vitro, eight *Lactobacillus* strains were isolated from Japanese fish and exhibited antioxidant properties, but notably these strains had differences in degrees of scavenging free radicals [26]. The reason for this discrepancy may be due to the different *Lactobacillus* strains used. It is absolutely imperative to test whether probiotic *Lactobacillus* had any antioxidant benefits when used in vivo; however, the related research findings have hitherto been scarcely available. Like all aerobic organisms, pigs particularly piglets are susceptible to the attack of ROS. It is well known that the living cell needs to control and maintain an adequate intracellular redox balance to function properly [27]. However, ROS at a high level is inclined to induce the obvious DNA and lipid oxidative injury with high concentrations of serum 8-OHdG and MDA. The 8-OHdG presently is the most popular biomarker for oxidative DNA damage, while MDA is a typical indicator of lipid peroxidation [28, 29]. Based on the above, in the current study, the LAB administration slightly decreased 8-OHdG generation and significantly declined MDA production throughout the study period, displaying less oxidative stress of piglets, which indicates antioxidant effects from LAB.

It is universally accepted that antioxidant enzymes including T-AOC, SOD, GSH-Px, and CAT are the important components of the antioxidant systems in the body, as they catalyze a variety of chemical reactions related to ROS and play a vital function for self-defense [30]. Research in humans have shown increased erythrocyte SOD and GPx activities as well as total antioxidant status in type 2 diabetic patients receiving probiotic yogurt containing *Lactobacillus acidophilus* La5 and *Bifidobacterium lactis* Bb12 [31]. Consistent with this, Rajput et al. [32] reported that the elevated T-AOC and SOD activities were observed in the serum and liver of ducks receiving 1 × 10^8^ cfu/kg probiotics in the diet. A study by Li et al. on probiotic-treated grass carps revealed the enhanced activities of serum and/or hepatic T-AOC, SOD, GSH-Px, and CAT [33]. Similarly, the present study confirmed these findings as our data showed that administration of LAB had higher activities of T-AOC and CAT in serum, together with increased activities of GSH-Px and CAT in the liver, but unexpectedly LAB had scarce effects on SOD activity in either serum or liver, suggesting that the activity of CAT is more easily affected by LAB than the activity of SOD. Anyhow, it was obvious that LAB administrated to piglets prior to weaning improved the antioxidant defense system of piglets during suckling and weaning phases. Nevertheless, the physiologic mechanism behind the oxidation-resistant ability of probiotics is not properly understood [34], and whether LAB has a direct or indirect action on enzymatic activity is unclear yet.

In general, the use of probiotics *Lactobacillus* for the prevention or therapy for gastrointestinal infection and disorder is their main application [35]. The gut is the largest immunologically competent organ in the body, and the action of *Lactobacillus* is related to the gastrointestinal tract. It is evidently indicated that *Lactobacillus* in healthy humans and animals could stimulate nonspecific immune response and facilitate immune protection. Many strains of *Lactobacillus* have been shown to promote intestinal IgA secretion in pigs [36]. Secretory IgA is the principal regulator of adaptive defenses on the intestinal mucosal surface to protect the intestinal epithelium from enteric toxins and pathogenic microorganisms, contributing to the maintenance of intestinal homeostasis [37]. In this study, sIgA concentration of both the jejunal and ileal mucosa generally increased with increasing age of piglets; more importantly, the intake of LAB further stimulated intestinal mucosal sIgA responses, which supports the former investigations [38, 39]. Therefore, it is conceivable that LAB is able to improve intestinal mucosal resistance to pathogens.

Intestinal inflammatory cytokines (e.g., IL-1*β*, IL-6, IL-12, TGF-*β*1, TNF-*α*, and IFN-*γ*) highly involved in the production of sIgA and released from activated T lymphocytes, macrophages, and dendritic cells play a central role in the immune defense system [40]. Besides, inflammatory mediators also include chemokines (e.g., IL-8, CX3CL1, and MIP3*α*) which are soluble chemotactic cytokines and exhibit regulatory functions in both innate and acquired immunity [41]. Concerning the immune response, *Lactobacillus*, for instance, has been reported to affect macrophage proliferation and cytokine and chemokine production [42]. However, to the best of our knowledge, *Lactobacillus* has contradictory regulation on the production and secretion of inflammatory cytokines and chemokines in different experimental models. A previous study showed that several probiotic strains induced the production of IL-6 and TNF-*α* in the jejunum and ileum of broilers [38]. Moreover, it has been shown that *Lactobacillus* was capable of inducing high secretion of IL-1*β* and IL-6 from dendritic cells and mononuclear cells and also induced a strong upregulation of IL-12 in vitro [43, 44]. In contrast, some investigators have found that IL-1*β*, IL-6, IL-12, IFN-*γ*, and IL-8 were inhibited in both the jejunal and ileal mucosa of probiotic-fed piglets and significantly lower IL-1*β* transcript was also found in probiotic-fed fish [45, 46]. Another independent study reported that TNF-*α* production was increased in the jejunal mucosa while it was suppressed in the ileal mucosa of suckling piglets when *Enterococcus faecium* EF1 was orally administrated [45]. These differences might be due to variations in animal species, ages of animals, and dose and mode of probiotic application, as well as time of application. Meanwhile, this is in part due to different probiotic strains that can elicit strain-dependent effects on the host and have diverse immunomodulatory effects in vivo [4]. In the present study, LAB augmented the production of an array of inflammatory cytokines and chemokines, such as IL-1*β*, IL-6, IL-12, CX3CL1, and MIP3*α* in the intestinal mucosa of suckling piglets, thereby triggering physiological inflammation and exhibiting immune function; besides, it is noteworthy that LAB also generated long-lasting alterations in cytokines and chemokines of the intestinal mucosa of piglets after weaning. Even though the different levels of immune modulation were induced in the jejunum compared with the ileum, this seems reasonable as the bacterial community between the jejunum and ileum is different, and they may have diversity in mucosal immune response [47].

Although these findings are encouraging, the molecular mechanism of major mediators linking inflammation is poorly defined in vivo. To this end, we evaluated the protein levels of intestinal COX2 and iNOS of piglets. It has been known that COX2, a highly inducible and immediate early response gene in macrophages, is transiently elevated in certain tissues in response to various immunologic or inflammatory stimuli such as cytokines. Considerable effort has also been made to identify if the activation of the iNOS gene is under cytokine control and is transcriptionally regulated. Upregulation of the COX2 and iNOS genes by inflammatory stimuli is found to occur at transcriptional and posttranscriptional levels [48]. In the current study, induction of the COX2 and iNOS genes by LAB shares a striking similarity with the production of inflammatory cytokines and chemokines, and these outcomes further verified and provided compelling evidence that LAB administration plays an important role in immunomodulatory effects in piglets.

In summary, the current study showed a beneficial role of LAB administration in suckling piglets, increasing the antistress capability to a certain extent, improving the antioxidant status, and stimulating the immune response. What is more, the oral administration of LAB during the suckling period had a lasting effect on antioxidation and immunostimulation of piglets after weaning. Our findings provide new insights into how probiotic LAB administration during the suckling phase not only plays an important role in a neonate and but also influences the future of the antioxidant defense system and intestinal immune system of a child, which provides information needed for the future use of LAB in human or swine to alleviate oxidative stress and promote intestinal health.

## Figures and Tables

**Table 1:** Effects of orally administrated *Lactobacillus delbrueckii* on serum biochemical parameters of piglets^1^.

Items	Day 21			Day 28			Day 49		
	CON	LAB	*P* value	CON	LAB	*P* value	CON	LAB	*P* value
TP, g/L	52.60 ± 2.83	55.13 ± 6.41	0.40	48.45 ± 4.23	49.92 ± 4.53	0.58	44.33 ± 2.22	44.95 ± 2.72	0.68
ALB, g/L	34.60 ± 5.01	35.15 ± 6.82	0.88	31.18 ± 3.87	33.27 ± 4.13	0.39	27.07 ± 1.66	26.93 ± 3.30	0.93
GLB, g/L	18.33 ± 1.86	20.00 ± 3.22	0.30	17.17 ± 1.72	17.33 ± 0.82	0.84	17.50 ± 2.07	18.67 ± 2.50	0.40
UN, mmol/L	2.82 ± 0.33	2.21 ± 0.47	0.02	2.66 ± 0.52	2.11 ± 0.14	0.02	2.27 ± 0.34	2.07 ± 0.31	0.33
GLU, mmol/L	6.98 ± 0.89	6.97 ± 0.86	0.99	5.92 ± 0.31	5.98 ± 0.39	0.77	4.61 ± 0.63	4.93 ± 0.63	0.39
ALP, U/L	945.55 ± 306.32	958.12 ± 309.49	0.95	578.20 ± 150.47	537.15 ± 90.27	0.58	394.08 ± 44.45	529.13 ± 76.86	<0.01

^1^Six piglets per treatment. CON = piglets in the negative control group; LAB = piglets in the group that was orally administrated with *Lactobacillus delbrueckii* during the suckling period.

**Table 2:** Effects of orally administrated *Lactobacillus delbrueckii* on serum antioxidant indices of piglets^1^.

Items	Day 21			Day 28			Day 49		
	CON	LAB	*P* value	CON	LAB	*P* value	CON	LAB	*P* value
T-AOC, U/mL	2.52 ± 0.09	3.32 ± 0.27	0.02	1.56 ± 0.18	1.53 ± 0.14	0.91	1.30 ± 0.09	1.26 ± 0.07	0.7
SOD, U/mL	28.69 ± 0.89	28.87 ± 0.24	0.86	28.94 ± 1.57	29.35 ± 0.66	0.81	29.61 ± 0.48	29.24 ± 0.37	0.56
GSH-Px, U/mL	450.83 ± 25.35	463.31 ± 21.25	0.71	454.82 ± 17.78	475.79 ± 22.38	0.48	451.99 ± 25.88	492.04 ± 17.52	0.23
CAT, U/mL	76.08 ± 3.51	86.46 ± 3.90	0.09	108.23 ± 12.04	111.18 ± 10.34	0.86	92.36 ± 3.27	114.40 ± 2.54	<0.01
MDA, nmol/mL	2.03 ± 0.26	1.29 ± 0.10	0.04	1.85 ± 0.23	1.28 ± 0.11	0.06	1.89 ± 0.13	0.97 ± 0.13	<0.01

^1^Six piglets per treatment. CON = piglets in the negative control group; LAB = piglets in the group that was orally administrated with *Lactobacillus delbrueckii* during the suckling period.

**Table 3:** Effects of orally administrated *Lactobacillus delbrueckii* on hepatic antioxidant indices of piglets^1^.

Items	Day 21			Day 28			Day 49		
	CON	LAB	*P* value	CON	LAB	*P* value	CON	LAB	*P* value
T-AOC, U/mg of protein	1.65 ± 0.14	1.69 ± 0.12	0.82	2.01 ± 0.25	2.07 ± 0.16	0.23	2.38 ± 0.06	2.41 ± 0.13	0.95
SOD, U/mg of protein	9.70 ± 0.38	9.16 ± 0.48	0.39	9.10 ± 1.14	8.26 ± 0.53	0.52	7.59 ± 0.07	7.32 ± 0.24	0.30
GSH-Px, U/mg of protein	408.32 ± 23.14	506.02 ± 24.01	0.02	509.13 ± 19.41	632.57 ± 29.79	0.01	579.09 ± 18.43	684.48 ± 32.75	0.02
CAT, U/mg of protein	50.51 ± 2.39	62.62 ± 2.09	<0.01	44.35 ± 2.74	48.94 ± 6.74	0.54	38.15 ± 1.32	40.66 ± 0.34	0.12
MDA, nmol/mg of protein	1.97 ± 0.31	1.17 ± 0.14	0.04	2.54 ± 0.49	1.14 ± 0.20	0.02	2.42 ± 0.20	1.50 ± 0.24	0.01

^1^Six piglets per treatment. CON = piglets in the negative control group; LAB = piglets in the group that was orally administrated with *Lactobacillus delbrueckii* during the suckling period.

## Data Availability

The data used to support the findings of this study are available from the corresponding author upon request.
